# Defining Pediatric Diarrhea in Low-Resource Settings

**DOI:** 10.1093/jpids/pix024

**Published:** 2017-05-15

**Authors:** Gillian A Levine, Judd L Walson, Hannah E Atlas, Laura M Lamberti, Patricia B Pavlinac

**Affiliations:** 1 Departments of Epidemiology,; 2 Global Health,; 3 Pediatrics, and; 4 Medicine (Infectious Disease), University of Washington, Seattle, Washington;; 5 Department of International Health, Johns Hopkins University, Baltimore, Maryland

**Keywords:** definitions, diarrhea, empiric basis, pediatrics

## Abstract

Differences in definitions of acute pediatric diarrhea result in variable estimates of morbidity and mortality, treatment coverage, and associations with risk factors and outcomes. We reviewed published literature and guidelines focused on acute pediatric diarrhea in low- and middle-income countries. Clinical guidelines most commonly defined diarrhea in terms of quantity of loose or watery stool with consideration of normal stool patterns, whereas research studies often relied exclusively on a quantitative definition. The most commonly used quantitative definition, ≥3 loose or watery stools in a 24-hour period, has been compared to gold standards of caregiver perception and visual inspection of stool, with variable agreement. Age, breast-feeding status, and setting (facility vs household-based) influence the performance of quantitative diarrhea definitions in children. Universal adoption of a set of valid gold standard definitions specifically aligned with various programmatic and research goals will lead to more accurate coverage estimates and better-informed resource prioritization.

Diarrhea is a leading cause of morbidity and mortality among infants and young children, particularly in low-resource settings [1–4]. Quantifying the diarrhea burden, consequences, and impact of interventions across settings requires an established operational definition of the syndrome of diarrhea. The ideal definition of childhood diarrhea should disaggregate clinically important diarrhea events from those that do not have acute or long-term consequences and serve as a proxy measure for the underlying pathological process of disease. In addition, the definition should be replicable across settings and easily adopted by persons with limited healthcare training in low- and middle-income countries.

Misclassification of diarrhea by nonspecific definitions may lead to inaccurate estimates of prevalence and treatment coverage, contributing to inappropriate prioritization of resource needs in already resource-constrained settings, and to erroneous attribution of exposures to diarrhea and of diarrhea to health outcomes. A community-based study in Nigeria comparing maternal report of diarrhea and number of loose stools to laboratory-observed consistency of a single stool sample found that diarrhea prevalence estimates varied by almost 100% (range in prevalence: 4.8%–9.5%) depending on which definition was used [5]. In a study conducted in Kenya, the odds ratios for the effect of lack of latrine ownership on subsequent diarrhea ranged from 2.2 to 13.5, and the statistical significance of the reported association depended on the definition of diarrhea used [6].

The current review aimed to summarize common research and clinical practice case-definitions of pediatric infectious acute diarrhea in high-burden, low-resource settings and to identify and describe the historical basis and possible limitations of these definitions.

## METHODS

A search of published literature and publically available guidelines for definitions of pediatric acute diarrhea in infants and children under 5 in high-burden settings in low- and middle- income countries was performed between June and August 2015. To summarize current definitions of pediatric diarrhea, we identified clinical practice recommendations and guidelines for pediatric diarrhea management focused primarily on low-resource settings. To summarize definitions used in research, we abstracted the definitions used in 2 recent diarrhea etiology studies and from individual studies included in 3 recently published systematic reviews related to diarrhea burden, the association between diarrhea and subsequent malnutrition, and the relationship between breastfeeding practices and diarrhea-associated morbidity and mortality [3, 4, 7–9]. Current programmatic definitions used in the Demographic and Health Surveys (DHS) and Multiple Indicator Cluster Surveys (MICS) were obtained from the DHS and MICS tools and interview guides available on program websites [10, 11]. Definitions were entered into a standardized summary table and organized according to category of intended use (clinical practice guidelines, programmatic, research).

To identify publications that compared and evaluated the validity and performance of different definitions, we searched MEDLINE to identify English language articles using combinations of search terms for “diarrhea,” “definition,” “case- definition,” “pediatric,” “acute,” and “classification,” with no date restriction. Reference lists from frequently cited publications were scanned to identify additionally relevant papers. Titles and abstracts were reviewed by one reviewer (G.A.L.) to identify articles relevant to the review objectives, and full text of select publications was subsequently reviewed in full by 2 reviewers (G.A.L. and P.B.P.).

To explore the origins and empiric basis of diarrhea definitions, we reviewed cited definition sources in the peer-reviewed literature, and in programmatic and clinical practice guidelines, tracing references to previously published sources until an original source without a previously published reference was identified. We excluded sources focused primarily on adult populations, well resourced settings, or noninfectious sources of diarrheal illness. Duration of diarrhea, diarrhea severity, and defining episodes of diarrhea were not the focus of this review.

## RESULTS

### Current Definitions and Historic Origins

We reviewed definitions from 7 clinical practice guidelines (all from the World Health Organization [WHO]), 2 programmatic documents, and 94 research publications (Supplementary Table 1). The definition of diarrhea in guidelines varied by source document and time. In 1988, the WHO published a consensus definition of diarrhea that combined a quantitative component and accounted for the normal stool patterns in breastfed infants: “*usually defined as the passage of three or more liquid motions (i.e. liquid enough to take the shape of the receiving container) during a 24-hr period. However, for exclusively breast-fed infants the definition is usually based upon what the mother considers to be diarrhea*” [12]. The current WHO definition does not deviate far from this earlier definition: “*the passage of 3 or more loose or liquid stools per day, or more frequently than is normal for the individual. Frequent passing of formed stools is not diarrhea, nor is the passing of loose, ‘pasty’ stools by breastfed babies*” [13]. World Health Organization-sponsored clinical practice recommendations for health workers use different variations of this definition [14], which are sometimes less specific, such as 3 or more loose stools in a 24-hour period without consideration of what is normal for the individual [15]. Other WHO-sponsored guidelines use definitions focused on an aberration from normal for the individual without a quantitative specification, such as caregiver report of diarrhea, or passage of loose or liquid stools more frequently than is normal for the child [16–18].

The DHS use caregiver report of diarrhea within a 2-week recall period to assess the period prevalence of diarrhea. Interviewers are prompted to define diarrhea for the caregiver if he/she is unfamiliar with the term. Specifically, the 2015 Phase 7 DHS survey asks, “*has (NAME) had diarrhea in the last 2 weeks?*” [19]. The instructions note that, “*if a respondent is not sure what we mean by diarrhea, tell her it means three or more loose or liquid stools per day.*” Likewise, diarrhea classified in the MICS is based on caregiver report of the child experiencing diarrhea in the previous 2 weeks. Similar to DHS, the interviewer training guide provides further explanation of the diarrhea definition to be used: “*3 or more loose or watery stools in the last 24 hours or more loose or watery stool than are normal for individual*” [20]. The MICS instructions also point out that breastfed babies may have multiple loose stools per day that are not diarrhea.

Definitions were abstracted from a total of 94 research studies: 2 recent etiology studies, 5 studies from a review of diarrhea and subsequent malnutrition, 69 from a review of diarrhea burden, and 18 from a review of the association between breastfeeding practices and diarrhea-associated morbidity and mortality [7–9, 21, 22]. Of the 94 research studies evaluated, 54% (*n* = 51) used a quantitative definition without any specification of an expected or normal pattern for the individual; 10% (*n* = 9) used a definition based on caregiver’s perception or report of whether the child had experienced diarrhea or experienced an aberration from their normal pattern, without requiring a quantitative cutpoint; 12% (*n* = 11) used a definition that was “either” quantitative “or” accounted for the caregiver’s perception of whether the child was experiencing diarrhea or an aberration from normal stool patterns; 8% (*n* = 8) used a definition that required “both” a quantitative cutpoint “and” the perception of a caregiver of diarrhea or an aberration from normal stool patterns; 2% (*n* = 2 studies) used a definition based on stool consistency; and 14% (*n* = 13) of studies did not define diarrhea (Supplementary Table 1). The most common quantitative definition used was 3 or more loose stools in a 24-hour period, which accounted for 65% (*n* = 33 studies) of the 51 studies that used quantitative definitions and 35% of all 94 research studies evaluated. A systematic review of randomized controlled trials reporting pediatric acute diarrhea as the primary outcome also reported 3 or more loose or watery stools in 24 hours, without consideration of normal stool patterns, to be the most common research definition [23].

### Comparing Diarrhea Definitions

To determine the validity of diarrhea definitions, a gold standard against which to compare other definitions and estimate sensitivity and specificity is needed. The most commonly cited source document for the diarrhea definition of 3 or more loose stools in a 24-hour period was published in 1991 and used caregiver perception of diarrhea as the gold standard [24]. In this community-based longitudinal surveillance study of 705 children under 5 years of age in Bangladesh, the authors compared the performance of definitions based on different quantitative cutpoints of numbers of stools to caregivers’ perceptions of diarrhea occurrence [24]. The authors explained that a caregiver would be familiar with an individual child’s normal stool patterns and would be uniquely able to identify an aberration from the normal pattern, which could be associated with a pathological or clinically relevant event. Based on a gold standard of the caregivers’ perceptions of diarrhea, a cutpoint of 3 or more loose or watery stools or 1 or more bloody stools in a 24-hour period achieved the best “balance” of sensitivity and specificity, with a sensitivity of 78% and specificity of 96%.

A quantitative definition, “*three or more liquid or semi-liquid stools [i.e. able to take the shape of a container] during the past 24 hrs*,” was considered the gold standard in a population-based survey of households with children under 3 years of age in Nigeria [5]. Compared with the quantitative gold standard, caregiver’s perception of diarrhea had 36% sensitivity; 64% of children were classified as having diarrhea by the quantitative definition but not by caregivers’ perception. The specificity of caregiver perception was 96%. Importantly, this study found that the concordance between caregiver’s report of diarrhea and the 3 or more loose stool definition varied by child age and feeding status. Caregivers were less likely to report that diarrhea was present when there had been 3 or more liquid or semiliquid stools in children under 2 months and in exclusively breastfed infants.

A third study among children under 3 years of age in Kenya compared caregiver report of diarrhea to a quantitative definition, “*three or more loose or watery stools in a 12-hour daylight period, or any single stool with blood, pus, or mucus,*” and to the gold standard of the observation of stool consistency by a trained observer [6]. Caregiver’s perception had 14% sensitivity and 95% specificity compared with the quantitative definition, and 79% sensitivity and 94% specificity compared with observation of stool consistency.

### Definition Considerations

The lower sensitivity of caregivers’ report compared with a quantitative definition may reflect an overestimation of true, clinically relevant, diarrhea when a quantitative definition is used, particularly in very young children or those who are exclusively breastfed. For example, caregivers may intuitively disaggregate more frequent loose stools attributed to breastfeeding or young age from those that result from illness. Young and exclusively breastfed infants have more frequent and looser/less-formed bowel movements than those who are older, mixed-fed, or formula-fed infants, as part of their normal stool patterns [25, 26]. Although breastfed infants and young infants defecate more frequently, especially in early life, the total volume of stool produced in breastfed and formula and mixed-fed infants is similar [27]. Given that exclusive breastfeeding is well established to be protective against diarrhea morbidity and mortality in young infants, high frequency of stools in breastfed infants may not be indicative of clinical risk [28].

Although caregivers may inherently disaggregate clinically relevant from normal loose stool patterns, they may more easily recall more severe disease and therefore miss mild diarrhea that may be captured by a quantitative definition [29, 30]. Caregiver recognition of diarrhea may be influenced by knowledge and public awareness campaigns leading to secular trends in estimates of diarrhea burden and treatment coverage [31]. Variability in diarrhea perception is also known to be associated with caregiver education, rural vs urban settings, and the language used to describe diarrhea [32–35]. In one region of Sierra Leone, local terminology for diarrhea included 11 different terms in 4 different local languages, each with a slightly different meaning and interpretation for type and severity, which could vary in clinical relevance [34]. These sources of variability in caregiver recall may result in erroneous estimates of diarrhea prevalence and calculation of treatment coverage from a denominator that is not reflective of true treatment need or that is not reproducible across settings [30, 36].

Quantitative diarrhea definitions have the advantage of being comparable across studies, regions, populations, and time. They may also be an adequate proxy for total volume of stool produced, and thus they may include an inherent measure of severity [37]. However, such definitions do not account for the normal stool patterns of individuals and may not always be associated with an abnormal or clinically relevant event. A further challenge of quantitative definitions is the reliance on caregivers to accurately report the number of stools over a finite period of time. Therefore, quantitative definitions remain vulnerable to challenges of reporting and recall bias due to low literacy and numeracy, language variability, and/or contextual relevance of a 24-hour period.

Visual inspection of stool for consistency may be more reproducible and comparable across studies than caregivers’ report and can be standardized with adequate training and quality-control techniques. However, classification based on visual inspection of a single stool does not account for frequency, total volume of stool produced, or the duration of the episode. Validity of diarrhea determination from a stool specimen may also be limited by challenges in ascertaining a sample at the relevant time point; by the time a sample is collected, the stool consistency may have changed and the diarrhea event may have ended. Logistical and financial constraints further limit the utility of visual stool specimen analysis, particularly in community settings. For example, visual inspection of stool would not be a logistically feasible addition to programmatic surveys, which are already complicated and labor intensive for data collectors and time consuming for respondents. Similar to a quantitative definition, this method also fails to account for the differences in individuals’ normal patterns or consistency, overall health of the child, or differences in patterns associated with age, diet, or feeding method.

The context in which the diarrhea definition is applied will also influence its validity. The accuracy of a definition is inevitably higher when applied to a population already conditioned on caregiver perception of illness. Therefore, the accuracy of a diarrhea definition in correctly classifying true, clinically relevant disease is likely higher when applied at a healthcare facility than applied in a community active surveillance setting. Diarrhea can be caused by a range of enteric infections and local and systemic inflammatory processes that may or may not be captured by any single diarrhea definition. For diarrhea caused by pathogens that produce profuse secretory diarrhea, such as rotavirus and *Vibrio cholerae*, a definition based on number of loose stools may be optimal. For diarrhea etiologies that result in less acute fluid loss but may be associated with chronic linear growth failure or other more chronic pathologies, such as *Cryptosporidium,* a definition that includes mother’s perception of child’s overall health may be warranted. Noninfectious causes of diarrhea, such as inflammatory bowel disease, recent administration of antibiotics, or other non-gastrointestinal infections such as malaria and dengue, for which diarrhea is a common secondary consequence, also complicate the accuracy of diarrhea definitions [38].

The validity of a diarrhea definition will also depend on the underlying pathophysiology the definition is intended to measure and the goal of its diagnosis. If identifying children with dehydrating diarrhea is the goal, as in the case of cross-sectional surveys aimed at assessing the coverage success of oral rehydration solution, a definition that maximizes the sensitivity and specificity of identifying cases at risk of progressing to dehydration should be used [39]. This definition may differ from one used in efforts to estimate attribution of diarrhea to other outcomes, such as environmental enteric dysfunction or malnutrition, where a definition should be optimized to disaggregate children with the highest and lowest likelihood of experiencing these sequelae.

## CONCLUSIONS

Differences in definitions of acute pediatric diarrhea limit accurate quantification of disease burden, treatment coverage, associations with risk factors and health outcomes, and intervention effects [6, 24, 40, 41]. As noted previously, the use of a quantitative definition alone may not always reflect a clinically relevant disease state. Until further validation studies are performed, research settings could consider systematic use of a definition that requires “both” that a minimum frequency threshold is met (≥3 loose/watery stools in a 24-hour period) “and” the perception by a caregiver that such an event constitutes an aberration from normal for the individual. Such a definition emphasizes the systematic integration of both the frequency measure and the consideration of caregivers’ perception of illness, a combination that, although recognized in early WHO guidelines, we found to be inconsistently applied in practice. Ultimately, a set of standardized definitions of diarrhea based on events of clinical relevance and that target potential pathways for intervention are needed for both research and programmatic evaluation. Biomarkers that represent underlying pathology, including markers of environmental enteric dysfunction, inflammation, microbial dysbiosis, as well as fluid loss, may aid in developing improved clinical definitions. In addition, studies to determine age and feeding status standardized stool frequencies among infants and young children in low-resource, high-burden settings may lead to more accurate definitions of diarrhea for specific subgroups. Universal adoption of a set of valid gold standard definitions specifically aligned with various programmatic and research goals will lead to more accurate estimates of coverage and better-informed resource prioritization.

## Supplementary Data

Supplementary materials are available at Journal of *the Pediatric Infectious Diseases Society* online.

## Supplementary Material

Levine_Diarrhea_Definitions_Table_Appendix

## Notes

***Acknowledgment.*** We thank the Univeristy of Washington Global Center for Integrated Health of Women, Adolescents and Children (Global WACh) and the Kenya Research and Training Center for their support during the preparation of this article.

***Disclaimer.*** The findings and conclusions in this paper are those of the authors and are not to be construed as official or as reflecting views of the University of Washington or other affiliated institutions. The funders had no role in the study design, data abstraction, data interpretation, or writing of the manuscript.

***Financial support.*** This work was supported by the Bill and Melinda Gates Foundation (grant number OPP1132140). P. B. P. receives funding from the Center for AIDS Research Enterics Study Team (grant number AI027757) and Global WACh.

***Potential conflicts of interest.*** All authors: No reported conflicts of interest.All authors have submitted the ICMJE Form for Disclosure of Potential Conflicts of Interest. Conflicts that the editors consider relevant to the content of the manuscript have been disclosed.
