# Supplementary material for: Defining Pediatric Diarrhea in Low-Resource Settings
Source: J Pediatric Infect Dis Soc. 2017 May 15;6(3):289–93. doi: 10.1093/jpids/pix024 (PMC5907861; doi:10.1093/jpids/pix024)
Supplement: Levine_Diarrhea_Definitions_Table_Appendix [file pix024_suppl_levine_diarrhea_definitions_table_appendix.docx]

Table 1. Definitions of pediatric acute diarrhea used in a selection of clinical guidelines, programmatic tools and published research from low-resource settings

|  | **Definition** | **Source** |
| --- | --- | --- |
| **World Health Organization Clinical Practice Guidelines** | | |
|  | ≥3 liquid motions (i.e. liquid enough to take the shape of the receiving container) during a 24 hour period. However, for exclusively breast-fed infants the definition is usually based upon what the mother considers to be diarrhea. | Persistent Diarrhoea In Children Living in Developing Countries, Memorandum, 1988^1^ |
|  | Increased frequency of loose stools as observed or reported by the mother; stool is watery or green, or contains mucus or blood | Managing newborn problems: A guide for doctors, nurses and midwives, 2003. ^2^ |
|  | Passage of unusually loose or watery stools, usually ≥3 times in a 24 hour period. However, it is the consistency of the stools rather than the number that is most important. Frequent passing of formed stools is not diarrhea. Babies fed only breast-milk often pass loose, “pasty” stools; this also is not diarrhea. Mothers usually know when their children have diarrhea and may provide useful working definitions in local situations. | The Treatment of Diarrhoea: A Manual for Physicians and Other Senior Health Workers, 2005^3^ |
|  | Passage of loose or liquid stools more frequently than is normal for the individual | Recommendations on the Management of Diarrhoea and Pneumonia in HIV-infected Infants and Children, 2010^4^ |
|  | Acute (watery) diarrhea: ≥3 loose stools per day. Dysentery: Blood mixed with stools. | Pocket Book of Hospital Care for Children, 2013^5^ |
|  | Passage of ≥3 loose or liquid stools per day, or more frequently than is normal for the individual. Frequent passing of formed stools is not diarrhea, nor is the passing of loose, ‘pasty’ stools by breastfed babies. | Diarrhoeal Disease Fact Sheet, 2013 ^6^ |
|  | *Infants < 2 months*: The stools have changed from usual pattern and are many and watery (more water than fecal matter). The normally frequent or semi-solid stools of a breastfed baby are not diarrhea.  *Children 2 months to 5 years*: Health worker assessment of “Does the child have diarrhea?” | Integrated Management of Childhood Illness Chart Booklet, 2014^7^ |
| **Programmatic Tools** | | |
|  | Caregiver report of diarrhea, or ≥3 loose or watery stools in 1 day, or more frequently than in formal for the child, noting that it is normal for exclusively breastfed babies to have ≥3 loose stools every day. Prompting from interviewer that diarrhea is ≥3 loose or watery stools per day, or more frequently than is normal for the child. | Multiple Indicator Cluster Surveys (MICS) Questionnaire for Children Under Five 2014^8,9^ |
|  | Caregiver report of diarrhea with prompting from interviewer that diarrhea is ≥3 loose or liquid stools per day. | Demographic and Health Survey (DHS) 2015^10^ |

| **Research ^i^** | | |
| --- | --- | --- |
| ***Frequency-based (n=51)*** | ≥4 stools in 24 hours with liquid or semi-liquid consistency or the presence of mucous and/or blood, reported by the mother or the caregiver and confirmed by examining physician | Cravioto, 1990 ^11^; Uribe, 1991 ^12^ |
|  | ≥4 or more loose, watery stools per day | Bhan, 1989 ^13^ ; Javaid, 1991 ^14^; Rahmathullah, 1991 ^15^; Kolsteren, 1997 ^16^ ; Oberhelman, 1999 ^17^; Tielsch, 2007 ^18^; Tielsch, 2007 ^19^ |
|  | ≥3 loose stools in a 24 hour period, or at least 1 loose stool with blood | Van de Hoek, 2002 ^20^ ; Baqui, 2003 ^21^ ; Jensen, 2004 ^22^; Genser, 2006 ^23^; Pathela, 2006 ^24^ ; Richard, 2014 ^25^ |
|  | ≥3 loose/watery/unformed stools in a 24 hours | Briend, 1988 ^26^; Aziz, 1990 ^27^; Lanata, 1991 ^28^; Oni, 1991^29^; Chowdhury, 1991 ^30^; Lanata, 1992 ^31^; Anand, 1994 ^32^; Barreto, 1994 ^33^; Cruz*,* 1994 ^34^; Ramakrishnan, 1995 ^35^; Santosham, 1995 ^36^; Dibley, 1996 ^37^; Gupta, 1996 ^38^; Linhares, 1996 ^39^; Venkatarao, 1996 ^40^; Yoon, 1996 ^41^; Espinoza, 1997 ^42^; Lopez-Alarcon, 1997 ^43^; Mirza*,* 1997 ^44^; Checkley, 1998 ^45^; Mulder-Sibanda, 1999 ^46^; Newman, 1999 ^47^ ; Ali, 2001 ^48^; Mahmud, 2001 ^49^; Moore, 2001 ^50^; Castro, 2003 ^51^; Haque, 2003 ^52^; Assis, 2005 ^53^; Strina, 2005 ^54^; Long, 2006 ^55^; Sazawal, 2007 ^56^ ; Mihrshahi, 2008 ^57^; Gladstone, 2010 ^58^ |
|  | ≥3 loose, watery or mucous stools or at least one bloody stool in 24 hours | Baqui*,* 1993 ^59^; Lie, 1993 ^60^ |
|  | ≥2 episodes of liquid stool in 24 hours | Sy, 2010 ^61^ |
| ***Care-giver perception/change from “normal” for individual (n=9)*** | Looser than usual stool consistency and increased frequency, as noted by their mothers or guardians | Ketsela, 1990 ^62^; Moy*,* 1991 ^63^; Lal, 1994 ^64^; Rosado*,* 1997 ^65^; Ibrahim, 1998 ^66^ Moraes*,* 2003 ^67^ |
|  | Mother impression of diarrhea based on what is normal for the child | Vieira, 2003 ^68^ |
|  | Caregiver’s report of diarrhea | Molbak, 1997 ^69^; Valentiner-Branth, 2001 ^70^ |
| ***Frequency-based OR based on care-giver perception/change from “normal” for individual (n=11)*** | ≥3 loose or liquid stools per day (or more frequent passage than is normal for the individual) | Naficy, 2000 ^71^ ; Banerjee, 2007 ^72^; Gupta, 2007 ^73^ |
|  | ≥3 unformed stools (or ≥1bloody) in a 24 hour period or maternal report of an increase in frequency or a decrease in consistency of stools in relation to the normal pattern of defecation | Abu Elyazeed, 1999 ^74^ |
|  | ≥3 unformed stools or the passage of unformed stools 2 times more than a particular child ordinarily used to pass per day | Yang, 1990 ^75^ |
|  | ≥3 watery stools in 24 hour or ≥2 bowl movements in addition to the usual daily pattern, with a decrease in stool consistency, or at least one bloody stool with a decreased consistency | Guerrero, 1998 ^76^ |
|  | ≥3 liquid stools, or when mothers stated that their child had diarrhea | Brunser, 1993 ^77^ |
|  | ≥3 unformed stools in a 24 hour period or mothers perception in the first 6 months of life | Qadri, 2007 ^78^ |
|  | ≥3 liquid stools in a 24 hour period or mother report in breast-fed infants | Mitra, 1997 ^79^ |
|  | ≥3 loose stools within 24 hours or any abnormal stools | Chen, 1981^80^; Jiraphongsa, 2005 ^81^ |
|  |  |  |
|  |  |  |
|  |  |  |
|  |  |  |
| ***Combination of frequency AND care-giver perception/change from “normal” for individual (n=8)*** | ≥3 abnormally loose stools within the previous 24 hours and accompanied by evidence of clinically significant dehydration, dysentery, or clinical decision to hospitalize^ii^ | Farag, 2012 ^82^ |
|  | ≥3 liquid or watery stools in 24 hours which is a marked change in the child's stool patterns as reported by the mother | Checkley, 2002 ^83^; Checkley, 2003 ^84^; Estrada-Garcia, 2009 ^85^ |
|  | ≥3 loose or liquid stools in any 24 hour period (for breastfed infants, also required a statement by mother that stools were more frequent or less formed than usual) | Clemens, 1999 ^86^ |
|  | ≥3 liquid or semiliquid stools and, in addition, was thought by his or her primary caretaker to have diarrhea | Bern, 2002 ^87^ |
|  | Mother perception of diarrhea and severe diarrhea defined as ≥5 loose stools per 24-hour period | Reller, 2003 ^88^ |
|  | An overt change in the child's normal stool pattern, characterized by an increase in the frequency (to ≥3 stools per 24 hour period) and a decrease in the consistency of stools to an unformed state | Ferreccio, 1991 ^89^ |
| ***Consistency-based (n=2)*** | Loose, semi-liquid, liquid, or watery stools with no visible blood or mucus | Molla, 1995 ^90^ |
|  | Liquid/semi liquid stools passed during 24 hour period | Brown, 1989 ^91^ |
| ***Undefined (n=13)*** | Undefined | Victora, 1987 ^92^; Cruz, 1989 ^93^; Mahmood, 1989 ^94^; Garrido, 1990 ^95^; Popkin, 1990 ^96^; Wright, 1991 ^97^; Molbak, 1994 ^98^; Arifeen, 2001 ^99^; Rao, 2003 ^100^; Bahl, 2005 ^101^; Macias-Carrillo, 2005 ^102^; Edmond, 2006 ^103^; Chhagan, 2009 ^104^ |

i. Definitions were grouped by theme, but separated when differences could plausibly result in variable classification of diarrhea (For example: “≥3 loose, watery or mucous stools or at least one bloody stool in 24 hours” vs. “≥3 loose, watery or mucous stools in 24 hours” were represented separately)

ii. Definition intended to capture cases of “moderate-to-severe” diarrhea

**References**

1. Persistent diarrhoea in children in developing countries: memorandum from a WHO meeting. Bulletin of the World Health Organization 1988;66:709-17.

2. Integrated Management of Pregnancy and Childbirth: Managing newborn problems: A guide for doctors,

nurses and midwives. Geneva, Switzerland: World Health Organization; 2003.

3. The Treatment of diarrhoea: a manual for physicians and other senior health workers, 4th rev. 4th ed. Geneva, Switzerland: World Health Organization; 2005.

4. WHO. WHO Recommendations on the management of diarrhoea and pneumonia in HIV-infected infants and children: IMCI. Geneva, Switzerland2010.

5. World Health Organization Pocket book of hospital care for children: guidelines for the management of common illnesses second edition Geneva, Switzerland WHO 2013.

6. Diarrhoeal disease Fact sheet N 330. 2013. (Accessed September 16, 2015, at <http://www.who.int/mediacentre/factsheets/fs330/en/>.)

7. Organization WH. Integrated Management of Childhood Illness Chart Booklet. Module 2: The sick young infant. March, 2014 ed. Geneva, Switzerland: World Health Organization; 2014.

8. Multiple Indicator Cluster Surveys: Questionnaire for Children Under Five. UNICEF, 2013. at <http://www.mics.unicef.org/tools>.)

9. Multiple Indicator Cluster Surveys: Instructions for Interviewers UNICEF, 2016. at <http://www.mics.unicef.org/tools>.)

10. USAID. Demographic and Health Surveys: Women's Health Module. Phase 7. 2015.

11. Cravioto A, Reyes RE, Trujillo F, et al. Risk of diarrhea during the first year of life associated with initial and subsequent colonization by specific enteropathogens. American journal of epidemiology 1990;131:886-904.

12. Uribe F, Hernandez R, Navarro A, Tello A, Benitez O, Cravioto A. [Patterns in the household acceptance of oral rehydration therapy in a cohort of rural mothers]. Boletin medico del Hospital Infantil de Mexico 1991;48:320-5.

13. Bhan MK, Bhandari N, Sazawal S, et al. Descriptive epidemiology of persistent diarrhoea among young children in rural northern India. Bulletin of the World Health Organization 1989;67:281-8.

14. Javaid N, Haschke F, Pietschnig B, et al. Interactions between infections, malnutrition and iron nutritional status in Pakistani infants. A longitudinal study. Acta paediatrica Scandinavica Supplement 1991;374:141-50.

15. Rahmathullah L, Underwood BA, Thulasiraj RD, Milton RC. Diarrhea, respiratory infections, and growth are not affected by a weekly low-dose vitamin A supplement: a masked, controlled field trial in children in southern India. Am J Clin Nutr 1991;54:568-77.

16. Kolsteren PW, Kusin JA, Kardjati S. Morbidity and growth performance of infants in Madura, Indonesia. Ann Trop Paediatr 1997;17:201-8.

17. Oberhelman RA, Gilman RH, Sheen P, et al. A placebo-controlled trial of Lactobacillus GG to prevent diarrhea in undernourished Peruvian children. The Journal of pediatrics 1999;134:15-20.

18. Tielsch JM, Khatry SK, Stoltzfus RJ, et al. Effect of daily zinc supplementation on child mortality in southern Nepal: a community-based, cluster randomised, placebo-controlled trial. Lancet 2007;370:1230-9.

19. Tielsch JM, Rahmathullah L, Thulasiraj RD, et al. Newborn vitamin A dosing reduces the case fatality but not incidence of common childhood morbidities in South India. The Journal of nutrition 2007;137:2470-4.

20. van der Hoek W, Feenstra SG, Konradsen F. Availability of irrigation water for domestic use in Pakistan: its impact on prevalence of diarrhoea and nutritional status of children. Journal of health, population, and nutrition 2002;20:77-84.

21. Baqui AH, Zaman K, Persson LA, et al. Simultaneous weekly supplementation of iron and zinc is associated with lower morbidity due to diarrhea and acute lower respiratory infection in Bangladeshi infants. The Journal of nutrition 2003;133:4150-7.

22. Jensen PK, Jayasinghe G, van der Hoek W, Cairncross S, Dalsgaard A. Is there an association between bacteriological drinking water quality and childhood diarrhoea in developing countries? Tropical medicine & international health : TM & IH 2004;9:1210-5.

23. Genser B, Strina A, Teles CA, Prado MS, Barreto ML. Risk factors for childhood diarrhea incidence: dynamic analysis of a longitudinal study. Epidemiology (Cambridge, Mass) 2006;17:658-67.

24. Pathela P, Zahid Hasan K, Roy E, Huq F, Kasem Siddique A, Bradley Sack R. Diarrheal illness in a cohort of children 0-2 years of age in rural Bangladesh: I. Incidence and risk factors. Acta Paediatr 2006;95:430-7.

25. Richard SA, Barrett LJ, Guerrant RL, Checkley W, Miller MA. Disease surveillance methods used in the 8-site MAL-ED cohort study. Clin Infect Dis. United States: Published by Oxford University Press on behalf of the Infectious Diseases Society of America 2014. This work is written by (a) US Government employee(s) and is in the public domain in the US.; 2014:S220-4.

26. Briend A, Wojtyniak B, Rowland MG. Breast feeding, nutritional state, and child survival in rural Bangladesh. British medical journal (Clinical research ed) 1988;296:879-82.

27. Aziz KM, Hoque BA, Hasan KZ, et al. Reduction in diarrhoeal diseases in children in rural Bangladesh by environmental and behavioural modifications. Transactions of the Royal Society of Tropical Medicine and Hygiene 1990;84:433-8.

28. Lanata CF, Black RE, Gilman RH, Lazo F, Del Aguila R. Epidemiologic, clinical, and laboratory characteristics of acute vs. persistent diarrhea in periurban Lima, Peru. Journal of pediatric gastroenterology and nutrition 1991;12:82-8.

29. Oni GA, Schumann DA, Oke EA. Diarrhoeal disease morbidity, risk factors and treatments in a low socioeconomic area of Ilorin, Kwara State, Nigeria. Journal of diarrhoeal diseases research 1991;9:250-7.

30. Chowdhury HR, Fauveau V, Yunus M, Zaman K, Briend A. Is acute watery diarrhoea an important cause of morbidity and mortality among rural Bangladeshi children? Transactions of the Royal Society of Tropical Medicine and Hygiene 1991;85:128-30.

31. Lanata CF, Black RE, Maurtua D, et al. Etiologic agents in acute vs persistent diarrhea in children under three years of age in peri-urban Lima, Peru. Acta Paediatr Suppl 1992;381:32-8.

32. Anand K, Sundaram KR, Lobo J, Kapoor SK. Are diarrheal incidence and malnutrition related in under five children? A longitudinal study in an area of poor sanitary conditions. Indian pediatrics 1994;31:943-8.

33. Barreto ML, Santos LM, Assis AM, et al. Effect of vitamin A supplementation on diarrhoea and acute lower-respiratory-tract infections in young children in Brazil. Lancet 1994;344:228-31.

34. Ramiro Cruz J, Cano F, Bartlett AV, Mendez H. Infection, diarrhea, and dysentery caused by Shigella species and Campylobacter jejuni among Guatemalan rural children. The Pediatric infectious disease journal 1994;13:216-23.

35. Ramakrishnan U, Latham MC, Abel R, Frongillo EA, Jr. Vitamin A supplementation and morbidity among preschool children in south India. Am J Clin Nutr 1995;61:1295-303.

36. Santosham M, Sack RB, Reid R, et al. Diarrhoeal diseases in the White Mountain Apaches: epidemiologic studies. Journal of diarrhoeal diseases research 1995;13:18-28.

37. Dibley MJ, Sadjimin T, Kjolhede CL, Moulton LH. Vitamin A supplementation fails to reduce incidence of acute respiratory illness and diarrhea in preschool-age Indonesian children. The Journal of nutrition 1996;126:434-42.

38. Gupta DN, Sircar BK, Sengupta PG, et al. Epidemiological and clinical profiles of acute invasive diarrhoea with special reference to mucoid episodes: a rural community-based longitudinal study. Transactions of the Royal Society of Tropical Medicine and Hygiene 1996;90:544-7.

39. Linhares AC, Gabbay YB, Mascarenhas JD, et al. Immunogenicity, safety and efficacy of tetravalent rhesus-human, reassortant rotavirus vaccine in Belem, Brazil. Bulletin of the World Health Organization 1996;74:491-500.

40. Venkatarao T, Ramakrishnan R, Nair NG, et al. Effect of vitamin A supplementation to mother and infant on morbidity in infancy. Indian pediatrics 1996;33:279-86.

41. Yoon PW, Black RE, Moulton LH, Becker S. Effect of not breastfeeding on the risk of diarrheal and respiratory mortality in children under 2 years of age in Metro Cebu, The Philippines. American journal of epidemiology 1996;143:1142-8.

42. Espinoza F, Paniagua M, Hallander H, Svensson L, Strannegard O. Rotavirus infections in young Nicaraguan children. The Pediatric infectious disease journal 1997;16:564-71.

43. Lopez-Alarcon M, Villalpando S, Fajardo A. Breast-feeding lowers the frequency and duration of acute respiratory infection and diarrhea in infants under six months of age. The Journal of nutrition 1997;127:436-43.

44. Mirza NM, Caulfield LE, Black RE, Macharia WM. Risk factors for diarrheal duration. American journal of epidemiology 1997;146:776-85.

45. Checkley W, Epstein LD, Gilman RH, Black RE, Cabrera L, Sterling CR. Effects of Cryptosporidium parvum infection in Peruvian children: growth faltering and subsequent catch-up growth. American journal of epidemiology 1998;148:497-506.

46. Mulder-Sibanda M, Sibanda-Mulder FS. Prolonged breastfeeding in Bangladesh: indicators of inadequate feeding practices or mothers' response to children's poor health? Public health 1999;113:65-8.

47. Newman RD, Sears CL, Moore SR, et al. Longitudinal study of Cryptosporidium infection in children in northeastern Brazil. The Journal of infectious diseases 1999;180:167-75.

48. Ali M, Asefaw T, Beyene H, Byass P, Hisabu M, Pedersen F. A community-based study of childhood morbidity in Tigray, Northern Ethiopia Ethiopian Journal of Health Development 2001;15:165-72.

49. Mahmud MA, Hossain MM, Huang DB, Habib M, DuPont HL. Sociodemographic, environmental and clinical risk factors for developing persistent diarrhoea among infants in a rural community of Egypt. Journal of health, population, and nutrition 2001;19:313-9.

50. Moore SR, Lima AA, Conaway MR, Schorling JB, Soares AM, Guerrant RL. Early childhood diarrhoea and helminthiases associate with long-term linear growth faltering. Int J Epidemiol 2001;30:1457-64.

51. Castro MX, Soares AM, Fonseca W, Rey LC, Guerrant RL, Lima AA. Common infectious diseases and skin test anergy in children from an urban slum in northeast Brazil. The Brazilian journal of infectious diseases : an official publication of the Brazilian Society of Infectious Diseases 2003;7:387-94.

52. Haque R, Mondal D, Kirkpatrick BD, et al. Epidemiologic and clinical characteristics of acute diarrhea with emphasis on Entamoeba histolytica infections in preschool children in an urban slum of Dhaka, Bangladesh. The American journal of tropical medicine and hygiene 2003;69:398-405.

53. Assis AM, Barreto ML, Santos LM, Fiaccone R, da Silva Gomes GS. Growth faltering in childhood related to diarrhea: a longitudinal community based study. European journal of clinical nutrition 2005;59:1317-23.

54. Strina A, Cairncross S, Prado MS, Teles CA, Barreto ML. Childhood diarrhoea symptoms, management and duration: observations from a longitudinal community study. Transactions of the Royal Society of Tropical Medicine and Hygiene 2005;99:407-16.

55. Long KZ, Montoya Y, Hertzmark E, Santos JI, Rosado JL. A double-blind, randomized, clinical trial of the effect of vitamin A and zinc supplementation on diarrheal disease and respiratory tract infections in children in Mexico City, Mexico. Am J Clin Nutr 2006;83:693-700.

56. Sazawal S, Dhingra U, Dhingra P, et al. Effects of fortified milk on morbidity in young children in north India: community based, randomised, double masked placebo controlled trial. BMJ (Clinical research ed) 2007;334:140.

57. Mihrshahi S, Oddy WH, Peat JK, Kabir I. Association between infant feeding patterns and diarrhoeal and respiratory illness: a cohort study in Chittagong, Bangladesh. International breastfeeding journal 2008;3:28.

58. Gladstone BP, Das AR, Rehman AM, et al. Burden of illness in the first 3 years of life in an Indian slum. J Trop Pediatr 2010;56:221-6.

59. Baqui AH, Black RE, Sack RB, Chowdhury HR, Yunus M, Siddique AK. Malnutrition, cell-mediated immune deficiency, and diarrhea: a community-based longitudinal study in rural Bangladeshi children. American journal of epidemiology 1993;137:355-65.

60. Lie C, Ying C, Wang EL, Brun T, Geissler C. Impact of large-dose vitamin A supplementation on childhood diarrhoea, respiratory disease and growth. European journal of clinical nutrition 1993;47:88-96.

61. Sy I, Handschumacher P, Wyss K, et al. [Variability of diarrheal diseases in children under 5 living in an urban setting: observations in Rufisque, Senegal]. Medecine tropicale : revue du Corps de sante colonial 2010;70:57-61.

62. Ketsela T, Asfaw M, Kebede D. Patterns of breast feeding in western Ethiopia and their relationship to acute diarrhoea in infants. J Trop Pediatr 1990;36:180-3.

63. Moy RJ, Booth IW, Choto RG, McNeish AS. Recurrent and persistent diarrhoea in a rural Zimbabwean community: a prospective study. J Trop Pediatr 1991;37:293-9.

64. Lal S. Surveillance of acute diarrhoeal diseases at village level for effective home management of diarrhoea. Indian journal of public health 1994;38:65-8.

65. Rosado JL, Lopez P, Munoz E, Martinez H, Allen LH. Zinc supplementation reduced morbidity, but neither zinc nor iron supplementation affected growth or body composition of Mexican preschoolers. Am J Clin Nutr 1997;65:13-9.

66. Ibrahim MM, Wall S, Persson LA. The impact of short stature on child morbidity in a rural African community. Ann Trop Paediatr 1998;18:145-54.

67. Moraes LR, Cancio JA, Cairncross S, Huttly S. Impact of drainage and sewerage on diarrhoea in poor urban areas in Salvador, Brazil. Transactions of the Royal Society of Tropical Medicine and Hygiene 2003;97:153-8.

68. Vieira GO, Silva LR, de OVT. [Child feeding and diarrhea morbidity]. Jornal de pediatria 2003;79:449-54.

69. Molbak K, Jensen H, Ingholt L, Aaby P. Risk factors for diarrheal disease incidence in early childhood: a community cohort study from Guinea-Bissau. American journal of epidemiology 1997;146:273-82.

70. Valentiner-Branth P, Steinsland H, Santos G, et al. Community-based controlled trial of dietary management of children with persistent diarrhea: sustained beneficial effect on ponderal and linear growth. Am J Clin Nutr 2001;73:968-74.

71. Naficy AB, Rao MR, Holmes JL, et al. Astrovirus diarrhea in Egyptian children. The Journal of infectious diseases 2000;182:685-90.

72. Banerjee I, Gladstone BP, Le Fevre AM, et al. Neonatal infection with G10P[11] rotavirus did not confer protection against subsequent rotavirus infection in a community cohort in Vellore, South India. The Journal of infectious diseases 2007;195:625-32.

73. Gupta DN, Rajendran K, Mondal SK, Ghosh S, Bhattacharya SK. Operational feasibility of implementing community-based zinc supplementation: impact on childhood diarrheal morbidity. The Pediatric infectious disease journal 2007;26:306-10.

74. Abu-Elyazeed R, Wierzba TF, Mourad AS, et al. Epidemiology of enterotoxigenic Escherichia coli diarrhea in a pediatric cohort in a periurban area of lower Egypt. The Journal of infectious diseases 1999;179:382-9.

75. Yang CR, Meng ZD, Wang X, Li YL, Zhang YX, Zhao QP. Diarrhoea surveillance in children aged under 5 years in a rural area of Hebei Province, China. Journal of diarrhoeal diseases research 1990;8:155-9.

76. Guerrero ML, Noel JS, Mitchell DK, et al. A prospective study of astrovirus diarrhea of infancy in Mexico City. The Pediatric infectious disease journal 1998;17:723-7.

77. Brunser O, Espinoza J, Araya M, Pacheco I, Cruchet S. Chronic iron intake and diarrhoeal disease in infants. A field study in a less-developed country. European journal of clinical nutrition 1993;47:317-26.

78. Qadri F, Saha A, Ahmed T, Al Tarique A, Begum YA, Svennerholm AM. Disease burden due to enterotoxigenic Escherichia coli in the first 2 years of life in an urban community in Bangladesh. Infection and immunity 2007;75:3961-8.

79. Mitra AK, Akramuzzaman SM, Fuchs GJ, Rahman MM, Mahalanabis D. Long-term oral supplementation with iron is not harmful for young children in a poor community of Bangladesh. The Journal of nutrition 1997;127:1451-5.

80. Chen LC, Huq E, Huffman SL. A prospective study of the risk of diarrheal diseases according to the nutritional status of children. American journal of epidemiology 1981;114:284-92.

81. Jiraphongsa C, Bresee JS, Pongsuwanna Y, et al. Epidemiology and burden of rotavirus diarrhea in Thailand: results of sentinel surveillance. The Journal of infectious diseases 2005;192 Suppl 1:S87-93.

82. Farag TH, Nasrin D, Wu Y, et al. Some epidemiologic, clinical, microbiologic, and organizational assumptions that influenced the design and performance of the Global Enteric Multicenter Study (GEMS). Clin Infect Dis. United States2012:S225-31.

83. Checkley W, Gilman RH, Black RE, et al. Effects of nutritional status on diarrhea in Peruvian children. The Journal of pediatrics 2002;140:210-8.

84. Checkley W, Epstein LD, Gilman RH, Cabrera L, Black RE. Effects of acute diarrhea on linear growth in Peruvian children. American journal of epidemiology 2003;157:166-75.

85. Estrada-Garcia T, Lopez-Saucedo C, Thompson-Bonilla R, et al. Association of diarrheagenic Escherichia coli Pathotypes with infection and diarrhea among Mexican children and association of atypical Enteropathogenic E. coli with acute diarrhea. Journal of clinical microbiology 2009;47:93-8.

86. Clemens J, Elyazeed RA, Rao M, et al. Early initiation of breastfeeding and the risk of infant diarrhea in rural Egypt. Pediatrics 1999;104:e3.

87. Bern C, Ortega Y, Checkley W, et al. Epidemiologic differences between cyclosporiasis and cryptosporidiosis in Peruvian children. Emerging infectious diseases 2002;8:581-5.

88. Reller ME, Mendoza CE, Lopez MB, et al. A randomized controlled trial of household-based flocculant-disinfectant drinking water treatment for diarrhea prevention in rural Guatemala. The American journal of tropical medicine and hygiene 2003;69:411-9.

89. Ferreccio C, Prado V, Ojeda A, et al. Epidemiologic patterns of acute diarrhea and endemic Shigella infections in children in a poor periurban setting in Santiago, Chile. American journal of epidemiology 1991;134:614-27.

90. Molla A, Bari A, Greenough WB, 3rd. Rice oral rehydration solution hastens recovery from dysentery. Journal of diarrhoeal diseases research 1995;13:8-11.

91. Brown KH, Black RE, Lopez de Romana G, Creed de Kanashiro H. Infant-feeding practices and their relationship with diarrheal and other diseases in Huascar (Lima), Peru. Pediatrics 1989;83:31-40.

92. Victora CG, Smith PG, Vaughan JP, et al. Evidence for protection by breast-feeding against infant deaths from infectious diseases in Brazil. Lancet 1987;2:319-22.

93. Cruz JR, Pareja G, Caceres P, Cano F, Chew F. [Acute and persistent diarrheal disease and its nutritional consequences in Guatemalan infants]. Archivos latinoamericanos de nutricion 1989;39:263-77.

94. Mahmood DA, Feachem RG, Huttly SR. Infant feeding and risk of severe diarrhoea in Basrah city, Iraq: a case-control study. Bulletin of the World Health Organization 1989;67:701-6.

95. Garrido F, Borges G, Cardenas V, Bobadilla JL, Ibarra J, Ruiz-Matus C. [Postneonatal mortality caused by diarrhea: a case-control study]. Salud publica de Mexico 1990;32:261-8.

96. Popkin BM, Adair L, Akin JS, Black R, Briscoe J, Flieger W. Breast-feeding and diarrheal morbidity. Pediatrics 1990;86:874-82.

97. Wright CE, el Alamy M, DuPont HL, et al. The role of home environment in infant diarrhea in rural Egypt. American journal of epidemiology 1991;134:887-94.

98. Molbak K, Gottschau A, Aaby P, Hojlyng N, Ingholt L, da Silva AP. Prolonged breast feeding, diarrhoeal disease, and survival of children in Guinea-Bissau. BMJ (Clinical research ed) 1994;308:1403-6.

99. Arifeen S, Black RE, Antelman G, Baqui A, Caulfield L, Becker S. Exclusive breastfeeding reduces acute respiratory infection and diarrhea deaths among infants in Dhaka slums. Pediatrics 2001;108:E67.

100. Rao MR, Abu-Elyazeed R, Savarino SJ, et al. High disease burden of diarrhea due to enterotoxigenic Escherichia coli among rural Egyptian infants and young children. Journal of clinical microbiology 2003;41:4862-4.

101. Bahl R, Frost C, Kirkwood BR, et al. Infant feeding patterns and risks of death and hospitalization in the first half of infancy: multicentre cohort study. Bulletin of the World Health Organization 2005;83:418-26.

102. Macias-Carrillo C, Franco-Marina F, Long-Dunlap K, Hernandez-Gaytan SI, Martinez-Lopez Y, Lopez-Cervantes M. [Breast feeding and the incidence of acute diarrhea during the first three months of life]. Salud publica de Mexico 2005;47:49-57.

103. Edmond KM, Zandoh C, Quigley MA, Amenga-Etego S, Owusu-Agyei S, Kirkwood BR. Delayed breastfeeding initiation increases risk of neonatal mortality. Pediatrics 2006;117:e380-6.

104. Chhagan MK, Van den Broeck J, Luabeya KK, Mpontshane N, Tucker KL, Bennish ML. Effect of micronutrient supplementation on diarrhoeal disease among stunted children in rural South Africa. European journal of clinical nutrition 2009;63:850-7.
